# Detailed Phytochemical Composition, Cyto-/Hepatotoxicity, and Antioxidant/Anti-Inflammatory Profile of Moroccan Spices: A Study on Coriander, Caraway, and Mystical Cumin

**DOI:** 10.3390/molecules29153485

**Published:** 2024-07-25

**Authors:** Hiba Bouzaid, Liliana Espírito Santo, Diana M. Ferreira, Susana Machado, Anabela S. G. Costa, Maria Inês Dias, Ricardo C. Calhelha, Lillian Barros, Oumaima Chater, Youssef Kandri Rodi, Faouzi Errachidi, Fouad Ouazzani Chahdi, Maria Beatriz P. P. Oliveira, Rita C. Alves

**Affiliations:** 1 Laboratory of Applied Organic Chemistry, Faculty of Sciences and Technology, University of Sidi Mohamed Ben Abdellah, B.P. 2202—Route d’Imouzzer, Fez 30000, Morocco; 2 REQUIMTE/LAQV, Department of Chemical Sciences, Faculty of Pharmacy, University of Porto, Rua de Jorge Viterbo Ferreira, n. º 228, 4050-313 Porto, Portugal; 3 Centro de Investigação de Montanha (CIMO), Instituto Politécnico de Bragança, Alameda Santa Apolónia, 5300-253 Bragança, Portugal; 4 Laboratório Associado para a Sustentabilidade e Tecnologia em Regiões de Montanha (SusTEC), Instituto Politécnico de Bragança, Campus de Santa Apolónia, 5300-253 Bragança, Portugal; 5 Laboratory of Functional Ecology and Environmental Engineering, Faculty of Sciences and Technology, University of Sidi Mohamed Ben Abdellah, Fez 30000, Morocco

**Keywords:** *Carum carvi* L., *Coriandrum sativum* L., *Ammodaucus leucotrichus* Cross. & Dur., nutritional profile, phenolic screening, cell-based assays

## Abstract

Coriander, caraway, and mystical cumin are famous for their aromatic properties and widely used in Moroccan cuisine. The nutritional/phytochemical composition of their seeds (used for food flavoring and preservation) were compared. Their antioxidant, anti-inflammatory, cytotoxic and hepatotoxic effects were also explored. The fat content was similar among the samples (13%), with monounsaturated fatty acids being predominant. The coriander and mystical cumin seeds were extremely rich in C18:1n9c (81 and 85%, respectively) while, in the caraway, C18:1n12 (25%) was found together with C18:1n9c (32%). The caraway seeds also presented a higher proportion of C18:2n6c (34%) than the other seeds (13 and 8%, correspondingly). γ-Tocotrienol was the major vitamin E form in all the samples. The caraway seeds contained double the amount of protein (~18%) compared to the other seeds (~8%) but, qualitatively, the amino acid profiles among all seeds were similar. The seeds were also rich in dietary fiber (40–53%); however, differences were found in their fiber profiles. Caraway showed the highest antioxidant profile and anti-inflammatory activity and an LC-DAD-ESI/MS^n^ analysis revealed great differences in the phenolic profiles of the samples. Cytotoxicity (NCI-H460, AGS, MCF-7, and CaCo_2_) and hepatotoxicity (RAW 264.7) were not observed. In sum, besides their flavoring/preservation properties, these seeds are also relevant source of bioactive compounds with health-promoting activities.

## 1. Introduction

Plants from the Apiaceae family (previously known as the Umbelliferae family) are cultivated worldwide for their food applications, being used as vegetables, spices, and herbs, and for different pharmaceutical purposes [1]. Spices and herbs, in particular, are globally used in the formulation of foodstuffs not only to enrich flavor and aroma but also because they present preservation properties that can be associated with their rich composition in bioactive compounds, such as polyphenols (e.g., flavones, tannins) and vitamins [2]. Moreover, their antioxidant, anti-inflammatory and anti-tumoral activities have also been described both in in vitro and in vivo studies [2,3].

Some plants from the Apiaceae family such as *Carum carvi*, *Coriandrum sativum*, and *Ammodaucus leucotrichus* are famous for their aromatic and medicinal properties [4], being traditionally used in Moroccan cuisine.

*Carum carvi* L., commonly known as caraway, is a globally cultivated plant renowned for its anti-inflammatory [5], spasmolytic, antioxidant [6], anti-hyperglycemic [7], carminative, and immunomodulatory properties. Caraway is a rich source of phytochemicals, such as monoterpenoids, flavonoids, and alkaloids [8], and its seeds are widely used as a spice in food [9] and in folk medicine [1] as a natural remedy for digestive, lactogenic, and carminative disorders [10].

*Coriandrum sativum* L., also known as coriander, is cultivated in different regions around the world and is used both as food seasoning and in traditional medicine to treat hyperglycemia and digestive complaints, such as indigestion, dysentery, and nausea [11,12]. Coriander is a rich source of fatty acids, including linolenic, palmitic, stearic, behenic, petroselinic, oleic, arachidic, linoleic, and myristic acids [2]. In addition, coriander seed extracts have demonstrated protective effects on the central nervous [13], gastrointestinal [11], and cardiovascular systems [14]. Additionally, it has shown antimicrobial properties against pathogens [15].

Finally, *Ammodaucus leucotrichus* Cross. & Dur. is an endemic plant from the North African region, especially Morocco and Algeria. It is known in some areas as “mystical cumin”, “hairy cumin”, or “Kamounn es-sofi” [16]. Traditionally, it has been used to treat digestive problems [16,17], cardiac diseases [17], and diabetes [16], as well as for it aphrodisiac and tonic properties [17]. Mystical cumin is also used as a flavoring agent and condiment in food seasoning [17]. It contains several bioactive compounds such as polyphenols, terpenoids, proteins, carotenoids, and lipids [17], and its seed extract has been reported to exhibit antibacterial [18,19,20], antioxidant [21], anti-inflammatory [22], and neuroprotective activities [23]. 

In the last years, an increasing awareness among food producers and consumers of the replacement of synthetic preservatives by natural ones has been observed. The search for natural compounds, spices, and herbs that can enhance the shelf life, stability, and organoleptic characteristics of food products has been a growing focus of investigation not only for scientific researchers, but also for food industries. The meat product industry, in particular, faces challenges of quality and safety, and effective processes need to be developed and employed to improve preservation in a more natural and safer way, reducing the adverse effects currently caused, for instance, by nitrates and nitrites commonly used in industrial meat products [24]. In this context, spices can be explored as alternative and natural food additives for meat product preservation, since they can prolong their shelf life, contributing to the inhibition of bacterial growth, reduction/elimination of foodborne pathogens [25], and flavor improvement. 

Most of the published studies about spices from the Apiaceae family are related to culinary applications [26], pharmacological activities [27], and phytochemical composition of their essential oils [28], but few information is yet available about the phenolic compounds [29,30], amino acids, and fatty acids [30] of the abovementioned plant seeds. 

In this work, we aim to highlight and compare the potential of caraway (*C. carvi*), coriander (*C. sativum*), and mystical cumin (*A. leucotrichus*) seeds as natural sources of nutritional and bioactive constituents for food preservation. These seeds, commonly used in meat product formulations [2,31], were evaluated for their antioxidant activities, anti-inflammatory potential, cytotoxicity, and hepatotoxicity, to determine the overall efficacy and safety of their use. This comprehensive analysis provides a detailed comparison of the bioactive properties and safety profiles of these three important seeds.

## 2. Results and Discussion

### 2.1. Proximate Composition of Seeds

Table 1 describes the nutritional profiles of the seeds under study. Total fat content was very similar among all of them (*p* > 0.05), about 13% (fw), while total minerals (ash content) were significantly higher in the caraway (7.7%). These seeds can also be highlighted for having had double the content of protein (19%) compared to coriander and mystical cumin (about 8–9% for both). In what concerns carbohydrates, all three seeds were very rich in dietary fiber (40–53%), but differences were found within the fiber profile. For instance, caraway was significantly richer in soluble fiber (6.6%, vs. 2.4 and 1.1%, for coriander and mystical cumin, respectively) and presented the lowest insoluble fiber content (34%), while coriander was the richest one (51%). In sum, nutritionally, these seeds are very good sources of fiber, especially insoluble fiber, whose consumption has been associated with several health benefits (e.g., improvement in digestive problems, arteriosclerosis, and blood fat levels and diabetes prevention, among others) [32,33,34]. Our results are in general agreement with those recently reported in [35], where the proximate composition of different Moroccan Apiaceae seeds was also studied and similar values of ash (5.8–6.6 g/100 g), lipids (10.6–12.9 g/100 g), proteins (8.2–13.1 g/100 g), and total carbohydrates (62.4–64.4 g/100 g) were found for the same seeds herein studied. However, it should be noted that, in comparison with our results, El-Assri et al. [35] reported a significantly lower protein content for *C. carvi* seeds (10.2%), while in our work found double the amount of protein (19.3%). Nevertheless, our result is corroborated by other studies. For instance, the USDA National Nutrient Database for Standard Reference [36] reported a similar protein content to ours for caraway seeds (19.8%). Still, for the same seeds, that database also describes similar contents to those presented in Table 1 for fats (14.6%), total carbohydrates (49.9%), and total fiber (38.0%). These differences among studies suggest that different factors can affect the proximate composition of seeds and eventually their taxonomic varieties (which were not known in this case) or edaphoclimatic conditions.

### 2.2. Total and Free Amino Acid Profiles

Being rich sources of protein, these seeds are also rich sources of different amino acids. A total amino acid profile embraces all the amino acids present in a sample regardless of their source (proteins, oligoproteins, or free amino acids). It was noticed that the sum of total amino acids (Table 2) of the different seeds was very similar to the crude protein values (Table 1) estimated by the Kjeldahl method—*C. sativum*: 10.6|9.4%; *C. carvi*: 18.6|19.3%; *A. leucotrichus*: 9.1|8.4%, respectively—which confirms that amino acids were the main sources of nitrogen in these samples.

As expected, based on their higher protein content (Table 1), the caraway seeds also contained the highest total amino acid content compared to coriander and mystical cumin (Table 2). However, qualitatively, the amino acid profiles of the three types of seeds were very similar. The major amino acid identified in all samples was glutamic acid (18–36 mg/g), followed by aspartic acid (9–22 mg/g). However, it is important to note that the total quantified amounts of these two amino acids (which were identified by chromatography after acid hydrolysis and derivatization) also accounted for the amounts of glutamine and asparagine that could have been originally present in the samples. This happens because glutamine and asparagine are deaminated and converted into respective carboxylic acids in acidic conditions. However, this deamination does not influence quantification of the total amino acid sum, since their molecular masses are quite similar (146 and 147 g/mol, for glutamine and glutamic acid, respectively; 132 and 133 g/mol, for asparagine and aspartic acid, correspondingly) [37]. Besides several health benefits reported in the literature [38], these amino acids have, above all, an important role in the umami taste [39,40]. These results are in accordance with those reported by Marcone et al. [41], who also described glutamine/glutamic acid as the major amino acid in caraway seeds, followed by the asparagine/aspartic acid pair.

As can be observed in Table 2, these seeds are also relevant sources of important amino acids such as arginine (7–14 mg/g), proline (4–9 mg/g), and branched-chain amino acids—leucine (5–12 mg/g), valine (4–9 mg/g), and isoleucine (4–9 mg/g)—which significantly contribute to cognitive functions and bodily physical performance [38]. In particular, arginine and branched-chain amino acids can promote reduction in blood pressure, contributing to the improvement in some cardiovascular injuries [38,42,43]. Recent investigations also elucidated that non-essential amino acids can be important anti-tumoral actors [44].

As mentioned above, the total amino acid profiles of the three types of seeds were very similar, although quantitatively, the caraway seeds presented higher contents of the individual amino acids, followed by coriander and the mystical cumin seeds, in that order. However, some exceptions should be highlighted as the following: the coriander and mystical cumin seeds presented no significant differences (*p* > 0.05) regarding glutamic acid, serine, tyrosine, and methionine contents (always being significantly lower (*p* < 0.05) than those of caraway); on the contrary, mystical cumin was a significantly better source (*p* < 0.05) of hydroxyproline (5 mg/g vs. 2 mg/g for coriander seeds, and 1 mg/g for caraway), being an important contributor to the composition of fibrillar collagen in the human body [45].

The different ratios between the hydroxyproline and the other amino acid contents could also be pointed out as interesting tools for discrimination studies among species, but a study with a higher number of samples from different geographical origins would be needed to obtain more accurate conclusions.

In what concerns the free amino acids (Table 3), i.e., amino acids that are not linked to other structures such as peptides or proteins, it was possible to observe significant differences (*p* < 0.05) between the profiles of the three samples. The caraway seeds contained the highest amount of free amino acids (8.17 mg/g); however, mystical cumin was distinguished by significantly higher (*p* < 0.05) values of free glutamine (~10-fold the amount found in the other two seeds). In general, the major free amino acids varied according to the samples (coriander: arginine > glutamic acid~lysine > aspartic acid > alanine; caraway: arginine > glutamic acid > asparagine > aspartic acid > alanine; mystical cumin: glutamine > asparagine > glutamic acid > proline > arginine), which highlights differences between the distinct species of seeds. Although in low amounts, these free amino acids can affect sensory taste receptors differently and contribute to the taste and flavor of spices [39]. A summary of the free contents presented in Table 3 are in similar ranges to those detected in other spices, such as cumin (8.1 mg/g) or cardamom (2.3 mg/g) [39].

### 2.3. Fatty Acid Composition and Vitamin E Profile

The fatty acid profiles of the analyzed seeds are presented in Table 4. The oils of these spices, especially coriander and mystical cumin, presented an extreme richness in monounsaturated fatty acids, namely oleic acid (81 and 85%, respectively), whose consumption has been highly associated with several health benefits, including a recognized protection against infections and inflammatory, immune, and cardiovascular diseases [46]. In turn, saturated fatty acids represented a minor fraction of the total fatty acids. Regarding the individual compounds, according to the results depicted in Table 4, the fatty acid profiles of coriander and mystical cumin were in general very similar (although with slight quantitative differences). Once more, caraway separated itself with a different profile, in which petroselinic acid appeared as one of the major fatty acids, which was not found in the other analyzed seeds. Also, caraway presented a significantly higher proportion of polyunsaturated fatty acids (more than 2- or 3-fold) compared with the remaining seeds. However, monounsaturated fatty acids were still predominant.

Petroselinic acid (C18:1n12), an isomer of oleic acid (C18:1n9), is a rare fatty acid but it can be found in many plants of the Apiaceae family. Several beneficial health properties have been attributed to this compound, namely, antidiabetic, antibacterial, and antifungal ones, and it has been used to reduce skin irritation, as a moisturizing agent, and to modulate lipid metabolism [47]. Levels ranging from 1 to 81.9% have been reported for coriander seed oil, depending on the plant variety, geographical origin, maturity index, and method of extraction [47]. In our case, petroselinic acid was only found in the caraway seeds at a percentage of 25%, which in is a similar range to those described in other studies [47,48]. For instance, Laribi et al. [49] demonstrated that in caraway seeds from three different ecotypes (Tunisian, German, and Egyptian), the content of petroselinic acid ranged from 29.46 to 31.12%, whereas oleic acid ranged from 28.72 to 30.0%. In general, the entire fatty acid profile of our caraway seeds almost overlaps with the one described by Kozłowska et al. [48] for samples acquired in a Polish store. In turn, Daga et al. [50] reported that oleic acid was the most abundant fatty acid in coriander (67%, which is more in accordance with our results) and caraway seeds (51%).

In what concerns the composition of tocopherols and tocotrienols (which combined constitute vitamin E), the seeds under study can also be considered good sources of these lipophilic antioxidant compounds (Table 4). The spices’ lipid fractions were screened for α-, β-, γ-, and δ-tocopherol, as well as for α-, β-, γ-, and δ-tocotrienol. The results are described in Table 4. β-Tocopherol, β-tocotrienol, and δ-tocopherol were not detected in any of the samples, with γ-tocotrienol being the major vitamin E form in all the seeds. Our findings are in agreement with those of Daga et al. [50], who reported the absence of δ-tocopherol in caraway seed oil, but, on the contrary, they found this vitamer in coriander seed oil. Curiously, Sriti et al. [51] reported the presence or absence of δ-tocopherol in the oil of coriander fruits depending on the variety (Canadian and Tunisian, respectively). Also, they reported γ-tocotrienol as the major vitamer in fruit oil, which is in accordance with our results for the seeds and is also corroborated by those of Wei et al. [52] for coriander fruit seeds (γ-tocotrienol > α-tocotrienol > α-tocopherol > δ-tocotrienol). In another study, Ziani et al. [53] described α-tocopherol as the only tocopherol present in mystical cumin, but in the present study, we also found γ-tocopherol.

Based on these findings, it was possible to understand that even within the same species, the lipid profile of the seeds varied widely. These differences could have been due to biotic conditions, including genetic factors, and exotic factors, such as environmental and edaphoclimatic ones.

### 2.4. Total Phenolics, Total Flavonoids, and Antioxidant Activity by Spectrophotometry

The extracts obtained from the coriander, caraway, and mystical cumin were also evaluated regarding their total phenolic content (TPC) and total flavonoid content (TFC). The results are presented in Table 5. TPC in the coriander seeds was 518 mg of gallic acid equivalents (GAE)/100 g, while in caraway, it reached 925 mg GAE/100 g, the highest value among all the seeds analyzed. Also, TFC was significantly (*p* < 0.05) higher in caraway seeds, while mystical cumin showed the lowest TFC (57 mg of catechin equivalents (CE)/100 g. It was already shown by Christova-Bagdassarian et al. [54] that caraway seeds have a higher level of total phenolics and total flavonoids (~26 mg GAE/100 g and ~12 mg CE/100 g dw, respectively) compared to coriander (17 mg GAE/100 g and 11 mg CE/100 g dw, correspondingly), which is consistent with our results. However, we estimated higher contents for both parameters (Table 5), which could have been due to the different methods of extraction employed. In fact, several studies report variations in TPC and TFC, according to the extraction method used. For instance, Btissam et al. [55] reported higher values using only water as an extracting solvent, while hydroalcoholic extraction led to lower yields of extraction in these seeds. Using similar extraction conditions to ours, Btissam et al. [55] described the TPC of ~90, 158, and 47 mg GAE/g dw, for coriander, caraway, and mystical cumin, respectively. According to the authors, the caraway seeds were also richer in total flavonoids (72 mg of quercetin equivalents (QuE)/g dw), followed by coriander (42 mg QuE/g dw), and mystical cumin (26 mg QuE/g dw) [55]. In another study, Gallo et al. [56] also showed the influence of extraction conditions, obtaining higher contents of total phenolics from coriander seeds by microwave-assisted extraction (82 mg GAE/100 g) compared to ultrasound-assisted (42 mg GAE/100 g).

Phenolics, including flavonoids, are considered the main antioxidant compounds of spices, with reducing, metal-chelating, and radical scavenging properties [57]. So, besides being used as natural food preservatives and flavoring, spices’ consumption can also display additional health benefits in relation to oxidative stress-related disorders (e.g., gastrointestinal disorders, neurodegenerative diseases, cardiovascular injuries, and cancers) [58]. To evaluate and compare the antioxidant capacity of coriander, caraway, and mystical cumin seeds, two different in vitro methods with complementary mechanisms of actions were undertaken, namely the DPPH^●^ scavenging assay and the ferric-reducing antioxidant power (FRAP) assay. The DPPH^●^ assay is commonly used as a screening method to evaluate the antiradical activity of a wide range of natural compounds. The radical can be neutralized by direct reduction (via electron transfer) or by radical quenching (via H atom transfer). In turn, in the FRAP assay, the reduction of Fe^3+^/2,4,6-tripyridyl-s-triazine complex occurs, which allows for the detection of compounds with redox potentials lower than 0.7 V (the redox potential of the complex), which is an acceptable screening method for the ability to maintain redox status in cells or tissues. However, the FRAP assay does not screen radical quenching by H transfer since only an electron transfer mechanism takes place [59]. As expected, owing to the seeds’ composition in total phenolics and total flavonoids, both models showed the antioxidant efficiency of the extracts, and the results are summarized in Table 5. Based on the previous results discussed, caraway showed the highest antioxidant average values, followed by mystical cumin and coriander (in that order), although no statistical differences (*p* > 0.05) were observed between caraway and mystical cumin in the FRAP assay (Table 5). Curiously, mystical cumin also presented a higher DPPH^●^ scavenging activity (~double) than coriander, although it contained ~3-fold fewer total flavonoids. This suggests that there are other compounds besides flavonoids in mystical cumin that are responsible for the antioxidant activity of these seeds. In addition, although in this study coriander showed a comparatively lower antioxidant activity in the DPPH^●^ and FRAP assays, a previous study by Martins et al. [60] showed that these seeds could achieve higher antioxidant values when using other methodologies. The authors expressed the antioxidant capacity as EC_50_ values (the extract concentration corresponding to 50% of the antioxidant activity) in which the higher values corresponded to lower reducing power/antioxidant potential. Indeed, Martins et al. [60] found significantly lower EC_50_ values using the TBARS (140 µg/mL) and β-carotene bleaching (~800 µg/mL) inhibition assays, in comparison to the results obtained by both DPPH^●^ and reducing power assays (~2000 µg/mL). This highlights the importance of using different methodologies to evaluate and provide a comprehensive understanding of the real antioxidant potential of a sample, since antioxidants can act through various mechanisms of action and no single assay can capture all those mechanisms.

In order to detail the phytochemical composition of these seeds and complement these results, the samples were further subjected to LC-DAD-ESI/MS^n^ analysis.

### 2.5. Phenolic Compound Quantification

Several phenolic compounds were tentatively identified in the samples by comparing their retention times, wavelengths of maximum absorption (λmax) in the UV–visible region, and MS data with those of available standards and literature data [53,61,62,63,64,65,66,67]. Significant differences were observed in the phenolic profiled of the three samples under study, with several compounds being found in only one or two of them. Overall, several phenolic compounds belonging to different classes (including phenolic acids, flavonoids, and isoflavonoids) were identified (Table 6).

It was possible to tentatively identify 18 phenolics in the coriander seeds, which showed, in comparison to caraway and mystical cumin seeds, to be especially rich in 3-*O*-caffeoylquinic acid (in both its *cis* and *trans* forms—peaks 1 and 2, respectively), quercetin derivatives [namely, quercetin-*O*-hexurunoside (peak 12), quercetin-*O*-hexosides (peaks 13 and 15), and quercetin-*O*-acetyl-hexosides (peaks 17, 19, and 28)], apigenin derivatives [apigenin-*C*-hexosyl-*C*-pentosides (peaks 3, 8, and 9) and an apigenin *C*-dihexoside (peak 6)], kaempherol derivatives [a kaempherol-*O*-hexurunoside (peak 22) and a kaempherol-*O*-acetylhexoside (peak 27)], and isorhamnetin derivatives [a isorhamnetin-*O*-deoxyhexosyl-hexoside (peak 20) and isorhamnetin-*O*-hexurunosides (peaks 23 and 25)]. Although they were not found in the current study, in a previous work, Martins et al. [60] also reported the presence of 3,5-*O*-dicaffeoylquinic acid, other quercetin derivatives (quercetin-3-*O*-rutinoside and quercetin-3-*O*-glucuronide), hesperetin-*O*-rutinoside, and caffeoyl N-tryptophan hexoside in coriander seeds. These differences could be due to the distinct extraction techniques used, as Martins et al. [60] employed a hydromethanolic extraction, whereas our study utilized a hydroethanolic one. Additionally, variations in seed composition due to different geographical origins and cultivation conditions cannot be ruled out.

Several studies have been reporting the health-promoting effects of such compounds belonging to the broader group of flavonoids (i.e., quercetin, apigenin, kaempherol, and isorhamnetin derivatives). Indeed, the antioxidant, antimicrobial, anti-inflammatory, anti-hypertensive, and anticancer properties of flavonoids have been widely described in the literature [68,69].

In turn, caraway seeds (with 14 identified phenolics) are a better source of chlorogenic acids, presenting eight compounds belonging to that family (esters of quinic acid with cinnamic acids [70], such as caffeoylquinic acids (peaks 1 and 2, also present in coriander seeds), feruloycaffeoylquinic acids (peaks 30, 31, and 32), a dicaffeoylquinic acid (peak 21), and a diferuloylquinic acid (peak 33)). The consumption of chlorogenic acids has been associated with several health benefits, in particular, in the context of cardiovascular diseases, type II diabetes, and inflammatory conditions [70].

Finally, mystical cumin presented a distinct profile composed of phenolic compounds that were not found in the other seeds, presenting an octyl gallate hexoside (peak 5), chicoric acid (peak 7), and a luteolin-*O*-hexoside (peak 16). In addition, like coriander, mystical cumin also presents an apigenin derivative (an apigenin-*C*-hexosyl-*C*-pentoside, peak 3) and a kaempherol derivative (a kaempherol-*O*-malonylhexoside, peak 26). This achievement seems to be in accordance with literature data, since other apigenin- and luteolin-derived compounds (e.g., apigenin-6,8-*C*-diglucoside, apigenin-*O*-(acetyl-hexoside), luteolin-7-*O*-glucoside, and luteolin-*O*-(malonyl-hexoside)) have already been found in the aerial parts of mystical cumin [53]. However, as aforementioned, in the seeds of this plant, we found relevant amounts of chicoric acid (1.7 mg/g dried extract), which also seems interesting in terms of health-promoting effects. In fact, antioxidant, anti-inflammatory, anti-obesity, antiviral, and neuroprotective effects have been described for this compound [71].

These large differences in the phenolic profile compositions of these seeds can partially justify the dissimilarities observed in their antioxidant activity. However, it should be noted that in the initial spectrophotometric screening (Table 5), only water was used as an extracting solvent. In that case, the yield of extraction should have been higher for more polar compounds, so the results of Table 5 and Table 6 cannot be directly compared, and instead, they should be seen as complementary. That justifies, indeed, why the proportions of total phenolics and total flavonoids found were different among the samples, when employing the different extraction solvents. In the last case, ethanol/water (80/20, *v*/*v*, Table 6), compounds with lower polarity were also extracted.

### 2.6. Anti-Inflammatory, Cytotoxic, and Hepatotoxic Activity: Cellular Assays

Macrophages play an important role in the immune system, especially in inflammatory processes. The RAW 264.7 cell line is widely used as a model to study inflammatory responses due to its role in producing pro-inflammatory mediators such as nitric oxide, and in this study, it was used to test the anti-inflammatory activity of the samples’ extracts. The following IC_50_ values were obtained: 286.5 ± 22.1 µg/mL for coriander (*C. sativum*), 34.4 ± 0.1 µg/mL for caraway (*C. carvi*), 102.3 ± 0.8 µg/mL for mystical cumin (*A. leucotrichus*), and 6.3 ± 0.4 µg/mL for the control (dexamethasone). All of these demonstrated the ability to inhibit nitric oxide formation, but significant differences were observed between the samples (*p* < 0.05), with caraway showing the highest anti-inflammatory activity. This could be due to its richness in compounds from the chlorogenic acid family (Table 6), since these compounds have already been previously described as potent anti-inflammatory agents [72,73]. For instance, chlorogenic acids have been shown to inhibit lipopolysaccharide-induced nitric oxide production and interleukin-1β expression by inhibiting JAK2/STAT3 activation in RAW 264.7 cells [73], thereby reducing inflammation. These results also highly correlate with those obtained for the antioxidant activity described in Table 5. In fact, antioxidant and anti-inflammatory activities are often interlinked, as oxidative stress can exacerbate inflammatory responses [74]. This correlation suggests that the phenolic compounds, particularly chlorogenic acids, present in these extracts not only scavenge free radicals and are able to transfer electrons, but also modulate inflammatory pathways, providing a dual mechanism of action.

Finally, in vitro assays were performed to evaluate cytotoxicity and hepatotoxicity with tumoral and non-tumoral cell lines, respectively, to explore the beneficial potential and/or safety of the seeds herein studied. The results showed that the samples’ extracts were not cytotoxic up to the maximum extract concentration tested (400 μg/mL) across the four cancer cell lines tested as the following: NCI-H460 (lung adenocarcinoma), AGS (gastric adenocarcinoma), MCF-7 (breast adenocarcinoma), and CaCo_2_ (colorectal adenocarcinoma).

Hepatotoxicity was tested in a porcine liver primary culture to assess the potential adverse effects of the extract, as these cells are more likely to reveal metabolic and toxic responses due to their active level of metabolization. Indeed, porcine liver cells are an excellent model for this purpose, offering a robust system to detect hepatotoxic effects that may not be as readily apparent in less metabolically active tissues. In this case, no hepatotoxicity was observed at the same concentrations mentioned above (up to 400 μg/mL).

For comparison, ellipticine (positive control) presented GI_50_ values between 1.01 ± 0.01 and 1.40 ± 0.10 μg/mL across all cell lines tested. This suggests that our extracts were non-toxic at the tested concentrations, highlighting their potential safety, but also that they might not promote an anti-tumoral effect (at least when used up to the tested concentrations). In contrast, the significantly lower GI_50_ values of ellipticine demonstrated its superior cytotoxic potency, promoting effects at a very low concentration. Although the observed lack of cytotoxicity and hepatotoxicity at the tested concentrations indicate a favorable safety profile for our extracts, these results also suggest that they may not have potent anti-tumoral properties or may require higher concentrations or even different extraction conditions to exhibit such effects. In a previous report, Lenzi et al. [75] investigated the in vitro anticancer properties of *A. leucotrichus* fruits from a wild population preparing ethanolic extracts by ultrasound-assisted maceration (that were further filtered and concentrated) in different human cell lines, two from hematological tumors and two from solid tumors: lymphoblast cells (TK6), promyelocytic cells (HL60), colorectal adenocarcinoma cells (DLD1), and neuroblastoma cells (SHSY5Y). The extract decreased cell viability in a dose-dependent manner in all analyzed cell lines after 24–26 h treatment, with inhibitory concentrations causing 50% of cell toxicity (IC_50_) of 0.22–0.39 mg/mL. In addition, the authors also reported that *A. leucotrichus* induced apoptosis and protected DNA from the toxicity of clastogen. The differences between our results and those reported by Lenzi et al. [75] can be justified by different factors. First, they used the fruit while our work was focused on the seeds. Also, the cell lines under study were different and may have had different susceptibilities. Regarding the method of extraction, Lenzi et al. [75] used ethanolic extracts prepared by ultrasound-assisted maceration, which might have enhanced the extraction efficiency and the yield of the bioactive compounds compared to the extraction procedure used in our study. In addition, while we used the sulforhodamine B assay, Lenzi et al. [75] employed other procedures, such as the Guava ViaCount Reagent (Luminex Corporation, Austin, LX, USA), containing 7-AAD or propidium iodide (PI) for suspension cells or the MTT [3-(4,5-dimethylthiazol-2-yl)-2,5-diphenyltetrazolium bromide] assay for adherent cells. 

So, it is important in future studies to consider different extraction methods that could lead to higher concentrations of bioactive compounds to further evaluate their potential safety and/or anti-tumoral potential in higher detail, since understanding these concentration-dependent effects is crucial to assessing the overall extracts’ potential applications. Additionally, further studies are needed to elucidate the complete mechanisms of action of these extracts. For instance, it would be important to explore the compounds’ bioavailability and metabolism, as well as their long-term safety and/or efficacy. For instance, simulating gastrointestinal digestion could provide relevant insights into how these compounds could be modified in the body and how these modifications could impact the activities herein studied.

## 3. Materials and Methods

### 3.1. Reagents, Standards, and Cells

Absolute ethanol, n-hexane (HPLC-grade), Kjeldahl tablets, sulfuric acid, sodium carbonate decahydrate (Na_2_CO_3_·10H_2_O), sodium hydroxide, anhydrous sodium sulfate (Na_2_SO_4_), formic acid, Folin–Ciocalteu reagent, catechin, gallic acid, 2,2-diphenyl-1-picrylhydrazyl (DPPH), a total dietary fiber assay kit, 2,4,6-tripyridyl-s-triazine (TPTZ), ferric chloride (FeCl_3_), Trolox, sodium nitrite (NaNO_2_), aluminum chloride (AlCl_3_), butylated hydroxytoluene (BHT), boron trifluoride in methanol (BF_3_), and the amino acids L-norvaline, L-aspartic acid (≥99.5%), L-glutamic acid (≥99.0%), L-asparagine (≥99.0%), L-glutamine (≥99.5%), L-alanine (≥99.5%), L-arginine monohydrochloride (≥99.0%), L-cystine (≥99.0%), L-valine (≥99.0%), L-threonine (≥99.0%), L-tyrosine (≥98.0%), L-leucine (≥98.0%), L-tryptophan (≥98.0%), L-lysine monohydrochloride (≥99.0%), glycine (≥99.0%), L-phenylalanine (≥98.0%), L-serine (≥99.0%), L-methionine (≥99.0%), L-isoleucine (≥99.0%), trans-4-hydroxy-L-proline (≥99.0%), L-proline (≥99.0%), and L-histidine monohydrochloride monohydrate (≥99.0%) were all acquired from Sigma-Aldrich (Darmstadt, Germany). 

The vitamin E standards (tocopherols and tocotrienols) and the internal standard tocol were acquired from Calbiochem (La Jolla, CA, USA). Potassium hydroxide and boric acid were acquired from Panreac (Barcelona, Spain). Dichloromethane was obtained from Riedel de Häen (Seelze, Germany). Ethanol (96%) was obtained from AGA (Prior Velho, Portugal). Phenolic compound standards (caffeic acid, apigenin-7-*O*-glucoside, chlorogenic acid, rosmarinic acid, hesperetin, naringenin, syringic acid, and quercetin-3-*O*-glucoside) were acquired from Extrasynthese (Genay, France). 

HPLC-grade methanol and acetonitrile were bought from Honeywell Riedel-de Haën (Seelze, Germany), n-hexane was from Carlo Erba, (Milan, Italy), and 1,4-dioxane from Honeywell Burdick & Jackson (Morris Plains, NJ, USA).

Water was treated in a Milli-Q water purification system (TGI Pure Water Systems, Greenville, SC, USA). All other reagents were of an analytical grade.

Human cell lines: CaCo_2_ (colorectal adenocarcinoma), AGS (gastric adenocarcinoma), and RAW 264.7 (murine macrophage) were provided from the ACACC: European Collection of Authenticated Cell Cultures; MCF-7 (breast adenocarcinoma) and NCI-H460 (lung adenocarcinoma) were acquired from the Leibniz Institute DSMZ (Braunschweig, Germany). Primary porcine liver cells were obtained and prepared as described in Section 3.12. 

For cell culture and related materials, Dulbecco’s modified Eagle’s medium (HyClone, Logan, UT, USA), Hank’s balanced salt solution (HBSS), and other components were procured from Gibco Invitrogen Life Technologies (Paisley, UK). A Griess reagent system kit was purchased from Promega (Madison, WI, USA). Other substances like thiamine, casamino acids, malt extract, and agar were supplied by Panreac AppliChem (Barcelona, Spain). Lipopolysaccharide (LPS), 2′,7′-dihydrodichlorofluorescein diacetate (DCFH-DA), 2,2′-azobis (2-amidinopropane) dihydrochloride (APPH), and sodium nitrate were obtained from Sigma-Aldrich (Barcelona, Spain).

### 3.2. Sample Preparation

The seeds of spices traditionally used in Moroccan cuisine for meat seasoning (*Coriandrum sativum* L., *Carum carvi* L., and the endemic North African *Ammodaucus leucotrichus*) were acquired at a Moroccan market. The samples were ground (<0.75 mm, GM200 GrindoMix; Retsch, Haan, Germany) and stored in the dark at room temperature until analysis. 

### 3.3. Moisture Content 

Moisture content was determined using an infrared moisture analyzer (DBS; KERN & SOHN GmbH, Balingen, Germany). A sample aliquot (3 g) was dried at 105 °C. Analyses were performed in triplicate and the results are expressed in % fresh weight (fw).

### 3.4. Ash Content

Total ash was quantified by incineration (Muffle Thermolyne 48,000; Electrothermal Engineering Ltd., Essex, UK) according to method 941.12 of the AOAC [76]. A sample aliquot (0.5 g) was heated gradually from 110 °C to 500–550 °C in a muffle until white ashes were obtained. The ash content was determined by the difference in mass contents before and after incineration. The analyses were performed in triplicate and the results are expressed in % fw.

### 3.5. Total Fat Content

Total fat content was determined using the Soxhlet method (991.36, AOAC [76]). Briefly, 5 g of spices were mixed with sand and anhydrous sodium sulfate. Then, the mixture was placed into cellulose cartridges. Sample extraction was carried out with petroleum ether during 8 h. After that, the residue was dried at 105 °C and weighed every 30 min until a constant mass value was obtained. The analyses were performed in triplicate and the results are expressed in % fw.

#### Study of the Lipid Fraction (Vitamin E and Fatty Acid Profiles)

A lipid fraction of the samples was obtained using a cold microextraction method as described by Melo et al. [77]. Briefly, 150 mg of sample was mixed with an antioxidant (0.1% BHT, 75 µL), the internal standard (0.1 mg/mL tocol, 50 µL), and 1 mL of ethanol. The mixture was vortexed for 30 min (Multi Reax EU; Heidolph, Berlin, Germany) and then 2 mL of n-hexane (HPLC-grade) was added. The mixture was shaken another 30 min, followed by the addition of 1 mL of 1% NaCl for salting out. After centrifugation at 5000 rpm (Heraeus Megafuge 16; Thermo Fisher Scientific, Waltham, MA, USA), the supernatant was collected, and the extraction repeated in the bottom phase. Both supernatants were combined and anhydrous sulphate was used to guarantee water absence. The extract was dried under a nitrogen stream until 1 mL was reached. This solution containing the lipid fraction was divided in two parts: 500 µL was directly injected into an HPLC system using the same conditions described by Alves et al. [78]; the remaining 500 µL was used for fatty acid analysis. In brief, n-hexane (HPLC-grade) was eliminated under a nitrogen stream and the residue was dissolved in 1 mL of dichloromethane and 2 mL of 0.5 M KOH in methanol. Transesterification was promoted at 100 °C for 10 min. The mixture was cooled in ice (5 min) before adding 2 mL of 14% BF_3_ in methanol (HPLC-grade). Methylation of free fatty acids occurred by heating the mixture at 100 °C for 30 min. After cooling in ice for a further5 min, 2 mL of deionized water and 2 mL of n-hexane (HPLC-grade) were added to the mixture to extract the fatty acid methyl esters [79]. The fatty acid profile was analyzed in a GC-2010 Plus gas chromatograph (Shimadzu, Tokyo, Japan) according to the exact conditions described by Melo et al. [77]. The analyses were performed in triplicate. Vitamin E content was expressed in mg/kg of seeds fw and fatty acid composition in relative %.

### 3.6. Total (Crude) Protein Content 

Total protein (TP) content was estimated following the Kjeldahl method (978.04, AOAC [76]) through the determination of total nitrogen (TN) content, which was then converted into crude protein using 5.18 as conversion factor [53]. Briefly, 0.5 g of spices was weighed on nitrogen-free paper and mixed with two Kjeldahl tablets (Darmstadt, Germany) and 20 mL of concentrated sulfuric acid (98%). Complete acid digestion was performed in an automatic digester (K-438; Büchi^®^, Büchi Labortechnik AG, Flawil, Switzerland), followed by distillation in a Kjeldahl distiller K-360 (Büchi^®^, Büchi Labortechnik AG, Flawil, Switzerland)), collection in a 4% boric acid solution, and titration with 0.2 M sulfuric acid. The analyses were performed in triplicate and the results are expressed in % fw.

#### Study of the Protein Fraction (Real Protein Estimation and Total/Free Amino Acid Profiles)

In order to estimate real protein, protein nitrogen (PN) (and subsequently real protein (RP) content) was determined by a similar protocol to the one used for TP, but with some modifications. Briefly, 200 mg of sample was mixed with 20 mL of deionized water and 4 mL of 15% (*w*/*v*) trichloroacetic acid. Filtration was carried out after 30 min of resting with a specific filter paper (low nitrogen content). The residue was washed twice with a solution of 15% trichloroacetic acid and then subjected to digestion, distillation, and titration as described above (Section 3.6) for TN determination. The RP content was estimated using 5.18 as a conversion factor [53]. 

Total amino acid profiles were analyzed as previously described [80,81]. Very briefly, acid hydrolysis of the samples (150 mg) was performed using 6 M HCl (3 mL), for 24 h, at 110 °C. For tryptophan quantification, an alkaline hydrolysis was additionally carried out (4 M KOH, 4 h, at 110 °C). The hydrolysates were then centrifuged (Heraeus Megafuge 16; Thermo Fisher Scientific, Waltham, MA, USA) and 50 µL of the respective supernatants was neutralized. The internal standard (10 µL, 2 mg/mL norvaline) was added before injection into an HPLC-DAD-FLD system, and amino acid derivatization occurred online with OPA/3-MPA and FMOC, exactly according to the previously published conditions [80].

In turn, to quantify free amino acids, the samples were milled into a fine powder (TM5; Thermomix, Vorwerk, Germany) and extracted for 30 min with deionized water (1:25, *w*/*v*), at room temperature, using a multi-rotator (Multi RS-60; Biosan Ltd., Riga, Latvia). After centrifugation (at 5000 rpm), the supernatant was collected, and the residue was re-extracted for 15 min. To 990 μL of the combined supernatants, 10 μL of 2 mg/mL norvaline was added. Online derivatization and chromatographic analysis were achieved as described above for total amino acid quantification [80]. The analyses were performed in triplicate and the results are expressed in % fw.

### 3.7. Carbohydrates and Dietary Fiber 

Total carbohydrate (TC) content was determined by the difference [82] using the following equation:

TC (% fw) = 100% (fw) − [Moisture (% fw) + Total Ash (% fw) + Total Fat (% fw) + Total Protein (% fw)]

Total dietary fiber (TDF) and insoluble dietary fiber (IDF) were analyzed according to the enzymatic–gravimetric AOAC methods 985.29 and 991.42 [76], respectively. Briefly, for total fiber, the sample was treated with different enzymes (α-amylase, amyloglucosidade, and protease) to simulate human digestion, breaking down starches and proteins, but leaving the fiber intact. The remaining residue was then precipitated with ethanol, filtered, dried, and weighed. Total dietary fiber content was calculated based on this residue. For insoluble fiber, an additional step of washing with ethanol and acetone to remove soluble materials was carried out. Soluble dietary fiber (SDF) was calculated by the difference. All the fiber analyses were performed in duplicate and the results are expressed in % fw.

Available carbohydrate (AC) content was determined by the difference [82] using the following equation:

AC (% fw) = 100% (fw) − [Moisture (% fw) + Total Ash (% fw) + Total Fat (% fw) + Total Protein (% fw) + Total fiber (% fw)]

### 3.8. Energy Value

Energy values were determined according to the European Parliament and Council [83] and are expressed in kcal/100 g of the sample and kJ/100 g of the sample, according to the following equations:

Energy value (kcal/100 g) = [(g of protein × 4 kcal) + (g of fat × 9 kcal) + (g of available carbohydrates × 4 kcal) + (g of fiber × 2 kcal)]

and

Energy value (kJ/100 g) = [(g of protein × 17 kJ) + (g of fat × 37 kJ) + (g of available carbohydrates × 17 kJ) + (g of fiber × 8 kJ)]

### 3.9. Estimation of Total Phenolics, Total Flavonoids, and Antioxidant Activity (Ferric-Reducing Antioxidant Power and DPPH^●^ Scavenging Activity) by Spectrophotometry

A sample aliquot (0.125 g) was extracted with 30 mL of water using a multi-rotator (Multi RS-60; Biosan Ltd., Riga, Latvia) for 60 min at 40 °C and 150 rpm. The extracts were prepared in triplicate, filtered, and analyzed regarding the total phenolic content (TPC) according to Costa et al. [59]. In brief, 150 µL of Folin–Ciocalteu reagent (1:10), and 120 µL of 7.5% (*m*/*v*) sodium carbonate, were added to 30 µL of liquid extract. After incubation (15 min, 45 °C), the mixture was left for 30 min at room temperature, protected from light. Absorbance was measured at 765 nm in a Synergy HT microplate reader (BioTek Instruments Inc., Santa Clara, CA, USA). A gallic acid calibration curve was prepared (5–100 mg/L, R^2^ = 0.9998). Analyses were carried out in triplicate and the results are expressed in mg of gallic acid equivalents (GAE) per 100 g of sample fw.

Total flavonoid content (TFC) was estimated according to Costa et al. [84]. Briefly, 1 mL of extract was mixed with 4 mL of deionized water and 300 µL of 25% sodium nitrite. After 5 min, at room temperature, 300 µL of 10% aluminum chloride was added. After 1 min, 2 mL of 1 M NaOH and 2.5 mL of deionized water were also added. Absorbance was read at 510 nm (Synergy HT GENS5; BioTek Instruments Inc., Santa Clara, CA, USA). A catechin calibration curve was prepared with linearity ranging between 2.5 and 400 mg/L, (R^2^ = 0.9998). Analyses were performed in triplicate and the results expressed in mg catechin equivalents (CE) per 100 g of sample fw.

Ferric-reducing antioxidant power (FRAP) was carried out according to the protocol previously described [84]. In brief, 270 µL of FRAP solution (composed of 0.3 M of acetate buffer, 10 mM of TPTZ, and 20 mM of ferric chloride) was added to 30 µL of extract. The mixture was incubated in the dark at 37 °C for 30 min, and absorbance was measured at 595 nm in a Synergy HT microplate reader (BioTek Instruments Inc., Santa Clara, CA, USA). Ferrous sulfate was used as calibration standard (y = 0.0022x − 0.0016, R^2^ = 0.9999, 25–500 mg/L). The analysis was performed in triplicate and the results are expressed as g of ferrous sulphate equivalents (FSE)/100 g of sample fw.

DPPH^●^ scavenging activity was determined as previously described [84]. Shortly, 30 μL of extract was mixed with 270 μL of a DPPH^●^ solution (6 × 10^−^^5^ M) and the reaction was measured every 2 min during 20 min to observe the kinetic reactions at 525 nm in a microplate reader (Synergy HT; BioTek Instruments Inc., Santa Clara, CA, USA). Trolox was used as calibration standard (y = −0.0068x + 0.5853, R^2^ = 0.9992, 5.62–175.34 mg/L). The analysis was performed in triplicate and the results are expressed in g of Trolox equivalents (TE)/100 g of sample fw.

### 3.10. Phenolic Compound Characterization by LC-DAD-ESI/MS^n^

For MS analysis, phenolic compounds were obtained by mixing 1 g of sample with 30 mL of ethanol/water (80/20, *v*/*v*), for 30 min at 25 °C, at 150 rpm (Multi RS-60; Biosan Ltd., Riga, Latvia). Then, the extracts were centrifuged at 5000 rpm (Heraeus Sepatech Labofuge Ae; Haraeus Instruments, Hanau, Germany) and the residue was re-extracted for 30 min. Finally, the supernatants were combined and filtered. Ethanol evaporation occurred under a nitrogen stream, followed by freeze drying, to obtain dried extracts. The phenolic profile was assessed by LC-DAD-ESI/MS^n^ (Dionex Ultimate 3000 UPLC; Thermo Scientific, San Jose, CA, USA). The compounds were analyzed according to Duan et al. [39]. The dried extracts were dissolved at a concentration of 10 mg/mL with methanol:water (80:20, *v*/*v*). Double online detection was performed using a diode array detector (monitoring at 280, 330, and 370 nm as the preferred wavelengths) and a mass spectrometer (MS). The MS detection was performed in negative mode, using a Linear Ion Trap LTQ XL mass spectrometer (Thermo Finnigan, San Jose, CA, USA) equipped with an ESI source.

The identification of the phenolic compounds was performed based on their retention time, UV spectra, and mass spectra, by comparison with analytical standards (when available) and literature data [61,62,63,64,65,66,67]. Data acquisition was carried out with an Xcalibur^®^ data system (Thermo Finnigan, San Jose, CA, USA). For quantitative analysis, a calibration curve for each available phenolic standard was constructed based on the UV signal. For those phenolic compounds for which a commercial standard was not available, the quantification was performed through the calibration curve of the most similar available standard. The results are expressed as the mg of compound/g of dried extract.

### 3.11. Anti-Inflammatory Activity

The dried extracts prepared in Section 3.10 were dissolved in water at a concentration of 8 mg/mL, which was further diluted (6.25–400 μg/mL). The RAW 264.7 mouse macrophage cell line was grown in DMEM medium supplemented with heat-inactivated fetal bovine serum (10%), glutamine, and antibiotics (37 °C, 5% CO_2_, under a humid atmosphere). Cells were detached with a cell scraper. An aliquot of the cell suspension of macrophages (300 μL), with a cell density of 5 × 10^5^ cells/mL and with a proportion of dead cells below 5% according to the trypan blue exclusion test, was placed in each well. The microplate was incubated for 24 h to allow an adequate adherence and multiplication of the cells. After that period, the cells were treated with different concentrations of extract (15 μL) and incubated for 1 h. Stimulation was performed with the addition of 30 μL of liposaccharide (LPS) solution (1 μg/mL) and incubated for an additional 24 h. Dexamethasone (50 µM) was used as a positive control and samples in the absence of LPS were used as a negative control. Quantification of nitric oxide was performed using a Griess reagent system kit (with nitrophenamide, ethylenediamine, and nitrite solutions) and through a nitrite calibration curve (100 µM sodium nitrite at 1.6 µM) prepared in a 96-well plate. The nitric oxide produced was determined by reading absorbances at 540 nm (ELX800 BioTek microplate reader; BioTek Instruments Inc., Santa Clara, CA, USA) and by comparison with the standard calibration curve. The results were calculated through the graphical representation of the percentage of inhibition of nitric oxide production vs. the sample concentration and expressed about the concentration of each of the extracts that caused the 50% inhibition of nitric oxide production (IC_50_) [85].

### 3.12. Cytotoxicity and Hepatotoxicity

The dried extracts prepared in Section 3.10 were dissolved in water at a concentration of 8 mg/mL, which was further diluted (6.25–400 μg/mL). To access cytotoxicity, different human tumor cell lines were used, namely (CaCo_2_, AGS, MCF-7, and NCI-H460). To investigate hepatotoxicity, a non-tumor cell culture was used (porcine liver primary cells), which was prepared from a freshly harvested porcine liver obtained from a local slaughterhouse, according to a procedure established by Souilem et al. [85]. The cells were maintained in RPMI-1640 medium supplemented with 10% fetal bovine serum, glutamine (2 mM), penicillin (100 U/mL), and streptomycin (100 mg/mL), except the porcine liver primary cells, which were maintained in DMEM medium supplemented with fetal bovine serum (10%), glutamine, and antibiotics. The culture flasks were incubated at 37 °C with 5% CO_2_, under a humid atmosphere. The cells were used only when they had 70 to 80% confluence. Each of the extract concentrations (10 μL) was incubated with the cell suspension (190 μL) of the cell lines tested in 96-well microplates for 72 h (incubation: 37 °C, 5% CO_2_, in a humid atmosphere), after checking cell adherence. All cell lines were tested at a density of 10,000 cells/well. Cold trichloroacetic acid (10% *w*/*v*; 100 μL) was added to stop the reaction and the microplate was incubated for 1 h at 4 °C. After that, it was washed with water and, after drying, a sulforhodamine B (SRB) solution (0.057%, *m*/*v*; 100 μL) was added and left to stand at room temperature for 30 min. To remove non-adhered SRB, plates were washed three times with a solution of acetic acid (1% *v*/*v*) and placed to dry. Finally, adherent SRB was solubilized with Tris (10 mM, 200 μL) and the absorbance at 540 nm was measured (BioTek ELX800 microplate reader; BioTek Instruments Inc., Santa Clara, CA, USA). The results are expressed in GI_50_ values (µg/mL), which correspond to the sample concentrations providing 50% of inhibition of cell growth. Ellipticine was used as a positive control [85].

### 3.13. Statistical Analysis

All analyses were performed in triplicate. Data are expressed as the mean ± standard deviation. One-way ANOVA followed by a post hoc Tukey HSD test was used for multiple comparisons among samples (IBM SPSS Statistics v. 28; IBM Corp., Armonk, NY, USA). The significance level was set at *p* < 0.05.

## 4. Conclusions

This study analyzed and compared the nutritional profiles and phytochemical compositions of coriander (*Coriandrum sativum*), caraway (*Carum carvi*), and mystical cumin (*Ammodaucus leucotrichus*) seeds, known for their aromatic properties and traditionally used in Moroccan cuisine for flavoring and food preservation. Additionally, their antioxidant, anti-inflammatory activities, and potential cytotoxic and hepatotoxic effects on different cell lines were investigated and compared.

Our findings demonstrated that all three types of seeds were rich in monounsaturated fat, protein, and dietary fiber and contained a wide range of phenolic compounds with relevant antioxidant and anti-inflammatory properties. However, differences were observed among the seeds’ compositions: petroselinic acid was found only in the caraway seeds while chicoric acid was present only in the mystical cumin seeds, and in turn, the coriander seeds were significantly richer in a wide variety of phenolic compounds. Additionally, the caraway seeds were the richest in total protein and soluble fiber.

The safety evaluation indicated no cytotoxicity or hepatotoxicity at the tested concentrations, confirming the suitability of these seeds for consumption. Moreover, all the seed extracts exhibited strong antioxidant and anti-inflammatory effects, with the caraway seeds having the most pronounced activities.

These findings highlight the potential health benefits of coriander, caraway, and mystical cumin seeds’ consumption beyond flavoring and food preservation applications. Overall, the three types of seeds showed to be valuable sources of bioactive compounds/phytochemicals with potential health-promoting properties.

In sum, this study provides an in-depth analysis of the nutritional, phytochemical, and bioactive profiles of coriander, caraway, and mystical cumin seeds, highlighting their potential health benefits which extend beyond their traditional uses in culinary practices. Our findings support their continued and expanded uses as functional ingredients that can contribute to overall health and well-being.

## Figures and Tables

**Table 1:** Proximate composition and energy values of *Coriandrum sativum*, *Carum carvi*, and *Ammodaucus leucotrichus* seeds.

Parameter	*Coriandrum sativum* (Coriander)	*Carum carvi* (Caraway)	*Ammodaucus leucotrichus* (Mystical Cumin)
Moisture (%)	7.40 ± 0.30 ^c^	8.60 ± 0.03 ^b^	12.20 ± 0.60 ^a^
Ash (%)	6.34 ± 0.04 ^c^	7.70 ± 0.04 ^a^	7.25 ± 0.04 ^b^
Crude protein (%)	9.40 ± 0.20 ^b^	19.30 ± 0.30 ^a^	8.44 ± 0.30 ^c^
True protein (%)	7.77 ± 0.26 ^b^	18.23 ± 1.76 ^a^	7.84 ± 0.29 ^b^
Fat (%)	13.62 ± 0.24 ^a^	13.88 ± 0.38 ^a^	13.10 ± 0.08 ^a^
Total carbohydrates (%)	63.30 ± 0.20 ^a^	50.50 ± 0.30 ^c^	59.00 ± 0.90 ^b^
Total dietary fiber (%)	53.21 ± 0.40 ^a^	40.26 ± 0.68 ^c^	47.55 ± 0.30 ^b^
Insoluble Fiber (%)	50.81 ± 0.34 ^a^	33.66 ± 0.22 ^c^	46.41 ± 0.03 ^b^
Soluble Fiber (%)	2.39 ± 0.05 ^b^	6.59 ± 0.46 ^a^	1.14 ± 0.27 ^c^
Remaining carbohydrates (%)	22.80 ± 0.60 ^b^	25.63 ± 0.77 ^a^	25.97 ± 1.16 ^a^
Energy value (kJ/100 g)	1476 ± 5 ^b^	1599 ± 10 ^a^	1450 ± 11 ^b^
Energy value (kcal/100 g)	358 ± 1 ^b^	385 ± 2 ^a^	351 ± 2 ^c^

The results are expressed as mean ± standard deviation (*n* = 3), in fresh weight. Different letters within each line represent significant differences at *p* < 0.05.

**Table 2:** Total amino acid content of *Coriandrum sativum*, *Carum carvi*, and *Ammodaucus leucotrichus* seeds (mg of amino acid/g of sample).

Amino Acid	*Coriandrum sativum* (Coriander)	*Carum carvi* (Caraway)	*Ammodaucus leucotrichus* (Mystical Cumin)
Essential or conditionally essential * amino acids			
* Arginine	6.90 ± 0.41 ^b^	14.08 ± 0.56 ^a^	6.34 ± 0.53 ^b^
* Histidine	3.60 ± 0.17 ^b^	6.24 ± 0.23 ^a^	2.52 ± 0.14 ^c^
Isoleucine	4.76 ± 0.31 ^b^	8.55 ± 0.38 ^a^	3.66 ± 0.14 ^c^
Leucine	6.64 ± 0.34 ^b^	12.49 ± 0.46 ^a^	5.38 ± 0.24 ^c^
Lysine	7.14 ± 0.49 ^b^	11.29 ± 1.32 ^a^	6.17 ± 0.20 ^b^
Methionine	0.80 ± 0.06 ^b^	2.24 ± 0.15 ^a^	0.79 ± 0.05 ^b^
Phenylalanine	4.68 ± 0.29 ^b^	8.65 ± 0.31 ^a^	3.82 ± 0.30 ^c^
Threonine	4.61 ± 0.33 ^b^	8.40 ± 0.09 ^a^	3.74 ± 0.16 ^c^
Tryptophan	0.97 ± 0.05 ^b^	1.34 ± 0.06 ^a^	0.60 ± 0.01 ^c^
Valine	5.54 ± 0.29 ^b^	9.62 ± 0.37 ^a^	4.24 ± 0.22 ^c^
Non-essential amino acids			
Alanine	5.64 ± 0.26 ^b^	10.10 ± 0.32 ^a^	4.22 ± 0.25 ^c^
Aspartic acid	12.94 ± 0.39 ^b^	22.08 ± 0.79 ^a^	9.58 ± 0.42 ^c^
Glutamic acid	19.35 ± 0.79 ^b^	36.02 ± 1.40 ^a^	18.48 ± 0.98 ^b^
Glycine	6.65 ± 0.29 ^b^	9.94 ± 0.61 ^a^	5.42 ± 0.31 ^c^
Hydroxyproline	2.17 ± 0.17 ^b^	1.26 ± 0.04 ^c^	4.84 ± 0.28 ^a^
Proline	5.55 ± 0.13 ^b^	9.25 ± 0.36 ^a^	4.37 ± 0.19 ^c^
Serine	5.98 ± 0.29 ^b^	10.22 ± 0.36 ^a^	5.39 ± 0.18 ^b^
Tyrosine	2.37 ± 0.10 ^b^	4.22 ± 0.10 ^a^	2.11 ± 0.13 ^b^
Ʃ Total amino acids	106.29 ± 4.97 ^b^	186.01 ± 7.49 ^a^	91.64 ± 4.00 ^c^

The results are expressed as mean ± standard deviation (*n* = 3), in fresh weight. Different letters within each line represent significant differences at *p* < 0.05.

**Table 3:** Free amino acid contents of *Coriandrum sativum*, *Carum carvi*, and *Ammodaucus leucotrichus* seeds (mg of amino acid/g of sample).

Amino Acid	*Coriandrum sativum* (Coriander)	*Carum carvi* (Caraway)	*Ammodaucus leucotrichus* (Mystical Cumin)
Essential or conditionally essential * amino acids			
* Arginine	0.33 ± 0.03 ^b^	1.66 ± 0.06 ^a^	0.25 ± 0.01 ^c^
* Histidine	0.04 ± 0.00 ^b^	0.11 ± 0.00 ^a^	0.03 ± 0.00 ^b^
Isoleucine	0.12 ± 0.01 ^b^	0.20 ± 0.01 ^a^	0.10 ± 0.00 ^c^
Leucine	0.15 ± 0.02 ^b^	0.23 ± 0.01 ^a^	0.13 ± 0.01 ^b^
Lysine	0.29 ± 0.02 ^a^	0.28 ± 0.01 ^a^	0.16 ± 0.00 ^b^
Methionine	0.07 ± 0.01 ^b^	0.12 ± 0.01 ^a^	0.05 ± 0.00 ^b^
Phenylalanine	0.09 ± 0.01 ^b^	0.20 ± 0.02 ^a^	0.08 ± 0.00 ^b^
Threonine	0.12 ± 0.01 ^b^	0.26 ± 0.01 ^a^	0.11 ± 0.00 ^b^
Tryptophan	0.08 ± 0.00 ^b^	0.11 ± 0.00 ^a^	0.04 ± 0.00 ^c^
Valine	0.11 ± 0.01 ^b^	0.28 ± 0.02 ^a^	0.12 ± 0.00 ^b^
Non-essential amino acids			
Alanine	0.23 ± 0.02 ^b^	0.66 ± 0.03 ^a^	0.16 ± 0.01 ^c^
Asparagine	0.16 ± 0.01 ^c^	1.03 ± 0.04 ^a^	0.37 ± 0.02 ^b^
Aspartic acid	0.24 ± 0.01 ^b^	0.76 ± 0.03 ^a^	0.20 ± 0.01 ^b^
Glutamine	0.10 ± 0.01 ^b^	0.08 ± 0.01 ^c^	1.06 ± 0.03 ^a^
Glutamic acid	0.29 ± 0.02 ^b^	1.28 ± 0.04 ^c^	0.32 ± 0.02 ^a^
Glycine	0.06 ± 0.00 ^b^	0.11 ± 0.01 ^a^	0.04 ± 0.00 ^c^
Hydroxyproline	0.01 ± 0.00 ^a^	0.01 ± 0.00 ^a^	0.01 ± 0.00 ^a^
Proline	0.12 ± 0.01 ^b^	0.29 ± 0.00 ^a^	0.28 ± 0.02 ^a^
Serine	0.13 ± 0.01 ^c^	0.35 ± 0.02 ^a^	0.17 ± 0.01 ^b^
Tyrosine	0.09 ± 0.01 ^b^	0.15 ± 0.01 ^a^	0.09 ± 0.01 ^b^
Ʃ Free amino acids	2.25 ± 0.29 ^c^	8.17 ± 0.29 ^a^	3.76 ± 0.08 ^b^

The results are expressed as mean ± standard deviation (*n* = 3), in fresh weight. Different letters within each line represent significant differences at *p* < 0.05.

**Table 4:** Lipid profiles of *Coriandrum sativum*, *Carum carvi*, and *Ammodaucus leucotrichus* seeds: fatty acid compositions (relative %) and vitamin E contents (µg/g of sample).

Fatty Acids (Relative %)	*Coriandrum sativum* (Coriander)	*Carum carvi* (Caraway)	*Ammodaucus leucotrichus* (Mystical Cumin)
C14:0	0.16 ± 0.01 ^a^	n.d.	0.08 ± 0.01 ^b^
C16:0	4.03 ± 0.03 ^c^	5.99 ± 0.22 ^a^	4.97 ± 0.38 ^b^
C16:1	0.22 ± 0.01 ^a^	0.12 ± 0.01 ^c^	0.15 ± 0.01 ^b^
C18:0	0.83 ± 0.06 ^c^	1.68 ± 0.04 ^a^	1.07 ± 0.10 ^b^
C18:1n9c	81.31 ± 0.10 ^b^	31.99 ± 1.35 ^c^	84.98 ± 0.74 ^a^
C18:1n12	n.d.	25.01 ± 1.55	n.d.
C18:2n6c	13.3 ± 0 0.14 ^b^	34.22 ± 0.45 ^a^	7.99 ± 0.35 ^c^
C18:3n3	0.16 ± 0.00 ^c^	0.41 ± 0.04 ^a^	0.32 ± 0.02 ^b^
C20:0	n.d.	0.26 ± 0.02	n.d.
C20:1n9	n.d.	n.d.	0.16 ± 0.01
C22:0	n.d.	0.33 ± 0.01 ^a^	0.29 ± 0.03 ^a^
Ʃ SFAs	5.02 ± 0.09 ^c^	8.26 ± 0.19 ^a^	6.41 ± 0.44 ^b^
Ʃ MUFAs	81.52 ± 0.01 ^b^	57.11 ± 0.25 ^b^	85.28 ± 0.74 ^a^
Ʃ PUFAs	13.46 ± 0.00 ^b^	34.63 ± 0.43 ^a^	8.31 ± 0.37 ^b^
Vitamin E profile (µg/g)			
α-Tocopherol	8.42 ± 0.40 ^c^	11.17 ± 0.53 ^b^	15.08 ± 1.07 ^a^
α-Tocotrienol	10.52 ± 0.26 ^b^	16.63 ± 0.22 ^a^	n.d.
β-Tocopherol	n.d.	n.d.	n.d.
β-Tocotrienol	n.d.	n.d.	n.d.
γ-Tocopherol	2.11 ± 0.12 ^a^	n.d.	2.26 ± 0.04 ^a^
γ-Tocotrienol	48.81 ± 0.78 ^c^	187.40 ± 1.61 ^a^	71.76 ± 3.45 ^b^
δ-Tocopherol	n.d.	n.d.	n.d.
δ-Tocotrienol	1.77 ± 0.05 ^b^	3.57 ± 0.19 ^a^	2.17 ± 0.10 ^b^
Total vitamin E	71.63 ± 0.76 ^c^	218.77 ± 2.23 ^a^	91.27 ± 4.20 ^b^

The results are expressed as mean ± standard deviation (*n* = 3). Different letters within each line represent significant differences at *p* < 0.05. C14:0, myristic acid; C16:0, palmitic acid; C16:1n7, palmitoleic acid; C18:0, octadecanoic acid; C18:1n9, oleic acid; C18:1n12, petroselinic acid; C18:2n6, linoleic acid; C18:3n3, α-linolenic acid; C20:0, arachidic acid; C20:1n9, eicosenoic acid; C22:0, docosanoic acid. SFAs, saturated fatty acids; MUFAs, monounsaturated fatty acids; PUFAs, polyunsaturated fatty acids; n.d., not detected. Vitamin E contents are expressed in fresh weight.

**Table 5:** Bioactive compounds and antioxidant activity of *Coriandrum sativum L.*, *Carum carvi L.*, and *Ammodaucus leucotrichus* seeds.

		*Coriandrum sativum* (Coriander)	*Carum carvi* (Caraway)	*Ammodaucus leucotrichus* (Mystical Cumin)
Bioactive compounds	Total phenolics (mg GAE/100 g)	517.8 ± 40.2 ^c^	925.4 ± 77.9 ^a^	689.0 ± 66.1 ^b^
	Total flavonoids (mg CE/100 g)	153.6 ± 14.7 ^b^	297.8 ± 32.3 ^a^	57.2 ± 7.9 ^c^
Antioxidant activity	Ferric-reducing antioxidant power (mg FSE/100 g) (µmol FSE/100 g)	4528 ± 449 ^b^	6802 ± 284 ^a^	6508 ± 616 ^a^
	DPPH^●^ inhibition (mg TE/100 g)	189.0 ± 16.0 ^c^	727.6 ± 70.2 ^a^	379.6 ± 70.2 ^b^

Results are expressed in fresh weight as mean ± standard deviation (*n* = 3). GAE, gallic acid equivalents; CE, catechin equivalents; FSE, ferrous sulphate equivalents; TE, Trolox equivalents. Different letters within each line denote significant differences at *p* < 0.05.

**Table 6:** Phenolic compositions and respective UV/MS data obtained from *Coriandrum sativum*, *Carum carvi*, and *Ammodaucus leucotrichus* seeds.

Peak	Rt (min)	λmax (nm)	[M − H]^−^ (*m*/*z*)	MS^n^ (*m*/*z*)	Tentative Identification	*Coriandrum sativum* (Coriander)	*Carum carvi* (Caraway)	*Ammodaucus leucotrichus* (Mystical Cumin)
1	6.91	325	353	191(100), 179(13), 173(15), 161(18), 135(8)	*cis* 3-*O*-Caffeoylquinic acid	3.079 ± 0.010 ^a^	0.616 ± 0.002 ^b^	n.d.
2	7.44	326	353	191(100), 179(11), 173(13), 161(14), 135(5)	*trans* 3-*O*-Caffeoylquinic acid	4.094 ± 0.005 ^a^	3.740 ± 0.032 ^b^	n.d.
3	9.93	333	593	575(8), 503(49), 473(100), 383(31), 353(40)	Apigenin-*C*-hexosyl-*C*-pentoside	0.099 ± 0.005 ^a^	n.d.	0.094 ± 0.000 ^a^
4	10.48	323	179	161(100), 135(24)	Caffeic acid	tr	0.233 ± 0.014	n.d.
5	13.17	342	443	281(25), 237(100)	Octyl gallate hexoside	n.d.	n.d.	0.661 ± 0.000
6	13.57	328	593	575(5), 503(5), 473(78), 431(100), 311(87)	Apigenin C-dihexoside	0.260 ± 0.012	n.d.	n.d.
7	13.95	330	473	311(100), 293(37)	Chicoric acid	n.d.	n.d.	1.687 ± 0.080
8	13.98	328	563	545(10), 503(34), 473(87), 443(100), 383(35), 353(24)	Apigenin-*C*-hexosyl-*C*-pentoside	0.130 ± 0.004	n.d.	n.d.
9	15.44	327	563	545(9), 503(29), 473(77), 443(100), 383(25), 353(14)	Apigenin-*C*-hexosyl-*C*-pentoside	0.010 ± 0.000	n.d.	n.d.
10	16.87	328	431	MS^2^: 341(23), 311(100); MS^3^: 283(100)	OH-Puerarin	0.529 ± 0.012	n.d.	n.d.
11	17.57	331	609	301(100)	Quercetin-3-*O*-rutinoside	n.d.	0.501 ± 0.002	n.d.
12	18.23	352	477	301(100)	Quercetin-*O*-hexurunoside	1.112 ± 0.011 ^a^	0.641 ± 0.005 ^b^	n.d.
13	18.64	346	463	301(100)	Quercetin-*O*-hexoside	1.284 ± 0.003 ^a^	0.494 ± 0.001 ^b^	n.d.
14	18.71	322	325	281(100), 193(4)	Ferulic acid conjugate	n.d.	n.d.	2.344 ± 0.120
15	19.06	343	463	301(100)	Quercetin-*O*-hexoside	0.620 ± 0.015	n.d.	n.d.
16	19.10	345	447	285(100)	Luteolin-*O*-hexoside	n.d.	n.d.	1.641 ± 0.020
17	20.35	352	505	301(100)	Quercetin-*O*-acetyl-hexoside	1.448 ± 0.021	n.d.	n.d.
18	20.51	326	527	365(100), 203(45), 185(1), 179(7), 135(5)	Di-caffeic acid derivative	n.d.	4.972 ± 0.130	n.d.
19	20.77	339	505	301(100)	Quercetin-*O*-acetyl-hexoside	1.431 ± 0.059	n.d.	n.d.
20	21.39	341	623	315(100)	Isorhamnetin-*O*-deoxyhexosyl-hexoside	0.672 ± 0.009	n.d.	n.d.
21	21.63	326	515	MS^2^: 353(100), 335(5), 229(3), 255(5), 203(6), 191(35), 179(12); MS^3^:173(100)	3,4-di-*O*-caffeoylquinic acid	n.d.	1.718 ± 0.082	n.d.
22	22.07	346	461	285(100)	Kaempherol-*O*-hexurunoside	0.566 ± 0.012	n.d.	n.d.
23	22.50	340	491	315(100)	Isorhamnetin-*O*-hexurunoside	0.881 ± 0.009	n.d.	n.d.
24	23.35	325	515	MS^2^: 353(100), 335(32), 179(12); MS^3^: 173(100)	1,4-di-*O*-caffeoylquinic acid	n.d.	0.791 ± 0.003	n.d.
25	23.48	348	491	315(100)	Isorhamnetin-*O*-hexurunoside	0.734 ± 0.033	n.d.	n.d.
26	23.93	341	533	489(54), 285(100)	Kaempherol-*O*-malonylhexoside	n.d.	n.d.	0.936 ± 0.040
27	24.84	340	489	285(100)	Kaempherol-*O*-acetylhexoside	0.890 ± 0.031	n.d.	n.d.
28	25.46	327	505	301(100)	Quercetin-*O*-acetyl-hexoside	0.545 ± 0.005	n.d.	n.d.
29	27.47	326	541	379(100), 203(10), 185(34)	Feruloyl N-tryptophan hexoside	n.d.	1.674 ± 0.034	n.d.
30	28.37	325	529	MS^2^:367(100), 353(1), 335(6); MS^3^:193(8), 191(4), 173(100)	*cis* 4-Feruloyl-5-caffeoylquinic acid	n.d.	0.310 ± 0.000	n.d.
31	28.63	326	529	MS^2^: 367(100), 353(1), 335(6); MS^3^:193(54), 191(5), 173(100)	*trans* 4-Feruloyl-5-caffeoylquinic acid	n.d.	0.657 ± 0.002	n.d.
32	29.25	325	529	MS^2^: 367(75), 353(100), 335(6); MS^3^:193(1), 191(23), 179(82), 173(100)	3-Feruloyl-4-caffeoylquinic acid	n.d.	0.301 ± 0.010	n.d.
33	32.15	324	543	MS^2^: 367(100), 349(6); MS^3^: 193(100), (34)	4,5-Diferuloylquinic acid	n.d.	0.259 ± 0.006	n.d.
					Total Phenolic Acids	7.173 ± 0.004 ^b^	15.270 ± 0.010 ^a^	4.692 ± 0.200 ^c^
					Total Flavonoids	11.365 ± 0.230 ^a^	1.636 ± 0.007 ^c^	2.671 ± 0.070 ^b^
					Total Isoflavonoids	0.529 ± 0.012	n.d.	n.d.
					Total Phenolic Compounds	19.067 ± 0.250 ^a^	16.906 ± 0.010 ^b^	7.363 ± 0.270 ^c^

Values are presented as the mean ± standard deviation (*n* = 3). The results are expressed as mg of compound per g of dried extract. Different letters within each line denote significant differences at *p* < 0.05. n.d., not detected; tr, traces.

## Data Availability

Data are contained within the article.
